# Impacts of Stroke on Muscle Perceptions and Relationships with the Motor and Functional Performance of the Lower Extremities

**DOI:** 10.3390/s21144740

**Published:** 2021-07-11

**Authors:** Wan-Ju Liu, Li-Fong Lin, Shang-Lin Chiang, Liang-Hsuan Lu, Chao-Ying Chen, Chueh-Ho Lin

**Affiliations:** 1 Master Program in Long-Term Care, College of Nursing, Taipei Medical University, Taipei 110, Taiwan; m437104007@tmu.edu.tw; 2 Department of Physical Medicine and Rehabilitation, Shuang Ho Hospital, Taipei Medical University, New Taipei City 235, Taiwan; 08168@s.tmu.edu.tw; 3 Department of Physical Medicine and Rehabilitation, Tri-Service General Hospital, School of Medicine, National Defense Medical Center, Taipei 114, Taiwan; andyyy520@yahoo.com.tw; 4 Department of Physical Therapy and Assistive Technology, National Yang-Ming University, Taipei 112, Taiwan; lianghsuan@mail.ndmctsgh.edu.tw; 5 Department of Rehabilitation Sciences, The Hong Kong Polytechnic University, Hong Kong 999077, China; Chao-Ying.Chen@polyu.edu.hk; 6 Center for Nursing and Healthcare Research in Clinical Practice Application, Wan Fang Hospital, Taipei Medical University, Taipei 116, Taiwan

**Keywords:** muscle perception, functional performance, stroke, ankle, muscle weakness

## Abstract

Stroke results in paretic limb disabilities, but few studies have investigated the impacts of stroke on muscle perception deficits in multiaxis movements and related functional changes. Therefore, this study aimed to investigate stroke-related changes in muscle perceptions using a multiaxis ankle haptic interface and analyze their relationships with various functions. Sixteen stroke patients and 22 healthy participants performed active reproduction tests in multiaxis movements involving the tibialis anterior (TA), extensor digitorum longus (EDL), peroneus longus, and flexor digitorum longus (FDL) of the ankle joint. The direction error (DE), absolute error (AE), and variable error (VE) were calculated. The lower extremity of Fugl-Meyer Assessment (FMA-LE), Barthel Index (BI), Postural Assessment Scale for Stroke Patients, Tinetti Performance-Oriented Mobility Assessment (POMA), and 10-m walk test (10MWT) were evaluated. VE of EDL for the paretic ankle was significantly lower than that for the nonparetic ankle (*p* = 0.009). AE of TA, EDL, and FDL and VE of EDL and FDL of muscle perceptions were significantly lower in healthy participants than in stroke patients (*p* < 0.05 for both). DE of TA for the paretic ankle was moderately correlated with FMA-LE (*r* = −0.509) and POMA (*r =* −0.619) scores. AE and VE of EDL for the paretic ankle were moderately correlated with the 10MWT score (*r =* 0.515 vs. 0.557). AE of FDL for the paretic ankle was also moderately correlated with BI (*r* = −0.562). This study indicated poorer accuracy and consistency in muscle perception for paretic ankles, which correlated with lower limb functions of stroke patients.

## 1. Introduction

Stroke is a common cause of death and also leads to disabilities, hospitalization, dementia, mild cognitive disorder, depression, fatigue, and poor quality of life [1]. Béjot et al. reported the annual incidence of stroke as >1 million, with a mortality rate of 13–35% in 2016 [1]. They also predicted that stroke incidence will increase and that more than 1.5 million Europeans could suffer from stroke each year by 2025 [1]. In addition, the cost of direct and indirect healthcare for people with cardiovascular disease was €34–€413, and the European healthcare system spends >€18 billion per year on stroke [2]. 

Stroke leads to central neurologic impairments and causes abnormal muscle tone and muscle weakness in the paretic limbs, which may impact perception inputs from muscles in movements and decrease motor and functional performances of lower extremities during daily activities [3,4,5,6,7,8,9]. Therefore, perception inputs can be affected when muscles contract and generate strength during dynamic functional activities of daily living for patients with stroke [10,11,12]. Gorst et al. revealed that sensory impairments and muscle weakness of the ankle can impact balance and ambulation performances following stroke and should be routinely assessed and monitored [8]. Recent studies also reported that perception impairment worsens over time in stroke patients [13,14], and 25–75% of such patients require assistance to perform daily activities [15,16]. Therefore, appropriate measurements that evaluate deficits in muscle perceptions using active joint reproduction tests in ankle movements are crucial and could provide true reflections of functional performances of the ankle joint during daily activities in post-stroke patients [17]. 

Several clinical tests are applied by clinical therapists and researchers to measure ankle perception with a single goniometer and calculate ankle-joint proprioceptive performances through active joint reproduction tests [17,18]. However, muscle perceptions and functional performances are sensed and performed under multiaxis movements (combining dorsiflexion and inversion, or plantarflexion and eversion movements) in ankle joints [19,20,21]. For example, the combination movements of the tibialis anterior (TA) are dorsiflexion and inversion of the ankle, the extensor digitorum longus (EDL) contributes to the eversion and dorsiflexion of the foot, the peroneus longus (PL) plays eversion and plantar flexion movements of the foot, and the flexor digitorum longus (FDL) contributes to foot plantarflexion and inversion [22]. Therefore, evaluation of muscle perceptions using plastic goniometers or a digital inclinometer on a single plane through active joint reproduction tests without following the muscle movement in multiple axes may not provide sufficient information for clinical therapists to understand stroke-related changes in muscle perception impairments of the ankle joint [17,18]. In addition, recent studies reported that the VICON system (motion capture technology and algorithms) and FASTRAK can be used to measure three-dimensional human movements, but due to the large testing field and high cost, these instruments cannot be used to simultaneously measure and track ankle movements [23,24]. Therefore, internet of multimedia things (IoMT) and wearable inertial measurement units (IMUs) with accelerometers, gyroscopes, and magnetometers have been developed to obtain three-dimensional motion kinematic data [23,25]. However, compared with the motion-capture (Mocap) system, results showed significant differences in the range of motion of the ankle joint between IMUs and Mocap [26]. Therefore, a convenient, low-cost, and reliable measurement tool for monitoring stroke-related changes in muscle perceptions in multiple movements of the paretic ankle is critical. Furthermore, few studies have investigated the relationships of muscle perception deficits with motor control or functional performances in the lower limbs of stroke patients. Correlation analysis of such muscle-based perception deficits with clinical motor and functional performances would help clinical therapists develop appropriate rehabilitation programs improve specific muscle perceptions and enhance the motor and functional performances for the daily lives of stroke patients. Therefore, the purposes of this study were to: (1) investigate stroke-related changes in muscle perceptions during multiple movements of the paretic ankle using a multiaxis ankle haptic interface and (2) analyze the relationships of muscle proprioception deficits with motor and functional performances of the paretic ankle joint in stroke patients. We hypothesized that paretic ankle has poorer accuracy and consistency in muscle perception and it is correlated with lower limb functions in stroke patients.

## 2. Materials and Methods

### 2.1. Design

**Figure 1:**
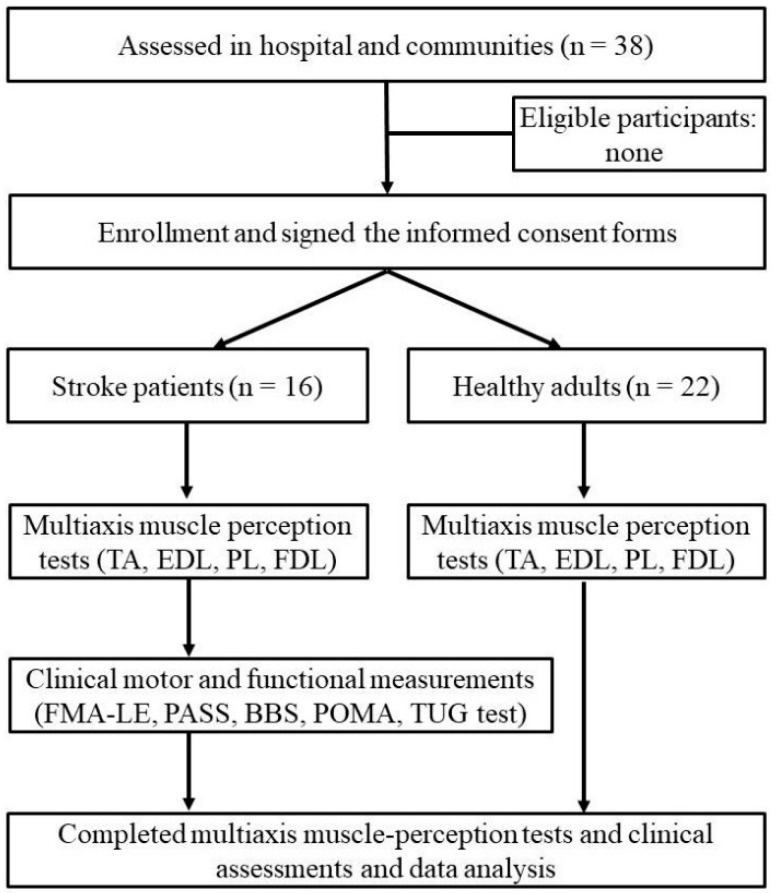
Flowchart for participant enrollment and outcome measurements.

This was a cross-sectional study. The flowchart in Figure 1 depicts participant enrollment. As a previous study had used active joint reproduction test to evaluate muscle perception in the sagittal and frontal planes, we used the same method to indicate muscle perceptions in this study [27]. Based on the electromyography (EMG) of the ankle musculature during gait cycle, the common key muscles involved in the activation of the ankle joint during gait included the TA, EDL, PL, FDL, and triceps surae [28]. Meanwhile, Magee reported that TA contributes to ankle inversion with dorsiflexion, EDL contributes to ankle eversion with dorsiflexion, the PL and brevis muscles contribute to ankle eversion with plantarflexion, and FDL mainly contributes to ankle inversion with plantarflexion [22]. Therefore, four specific test positions within functional ranges were selected, and active joint reproduction tests were performed within the range of functions for daily activities to infer each muscle perception of the ankle joint [20,29,30,31]. Test position I was 10° inversion with 10° dorsiflexion, which was used to represent perception by TA; test position II was 10° eversion with 10° dorsiflexion, which was used to represent perception by EDL; test position III was 10° eversion with 10° plantarflexion, which was used to represent perception by PL; and test position IV was 10° inversion with 10° plantarflexion, which was used to represent perception by FDL. 

### 2.2. Participants

We calculated the required sample size according to a previous similar study [17] using G*Power (version 3.1.9.2, Heinrich-Heine-Universität, Düsseldorf, Germany). To satisfy an α level of 0.05 and a power of 0.8, a minimum sample of 14 participants was required for each of the two groups. We enrolled 16 chronic stroke patients and 22 age-matched healthy participants in this study. Participant characteristics are shown in Table 1. The inclusion criteria for stroke patients were as follows: (1) diagnosis of a unilateral hemorrhagic or thrombotic stroke, (2) no other diagnoses accounting for motor performance deficits in either ankle joint, (3) a duration of at least 6 months after stroke [32], (4) Brunnstrom stage ≥4 (patients can move their paretic ankles in an isolated manner), (5) a mini-mental state examination score of ≥24 [33], (6) a modified Ashworth score of <3 for ankle plantarflexion, dorsiflexion, inversion, and eversion [34], and (7) ability to perform full range of motions in both ankle joints. Exclusion criteria were as follows: (1) stroke involving bilateral hemispheres, (2) inability to perform ankle joint movements (including plantarflexion, dorsiflexion, inversion, and eversion) because of abnormal muscle spasticity, (3) other orthopedic diseases or trauma affecting ankle motion and functional performance, (4) severe joint contracture or apraxia of bilateral ankle joints, and (5) pain or discomfort during tests in the study period. All participants provided informed consent for inclusion before participation in the study. The study was conducted in accordance with the Declaration of Helsinki, and the protocol was approved by the institutional review board of Taipei Medical University (no. N201609038).

### 2.3. Instruments

**Figure 2:**
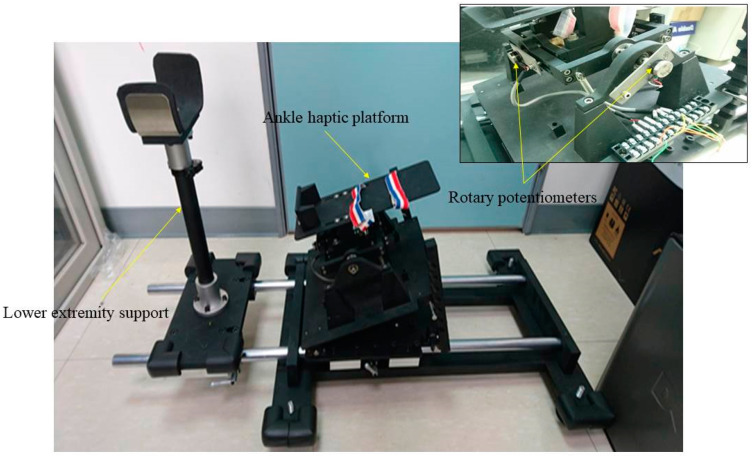
The multiaxis ankle-joint-perception measurement system and its components.

A multiaxis ankle-joint-perception measurement system with excellent validity was developed to measure dual-axis motions (in the sagittal and frontal planes) in degrees of the ankle joint [35]. This system comprises a tilting adjustable ankle haptic platform with two rotary potentiometers (100 K ± 0.05% Ω, Rmax, Taipei, Taiwan) (Figure 2). 

**Figure 3:**
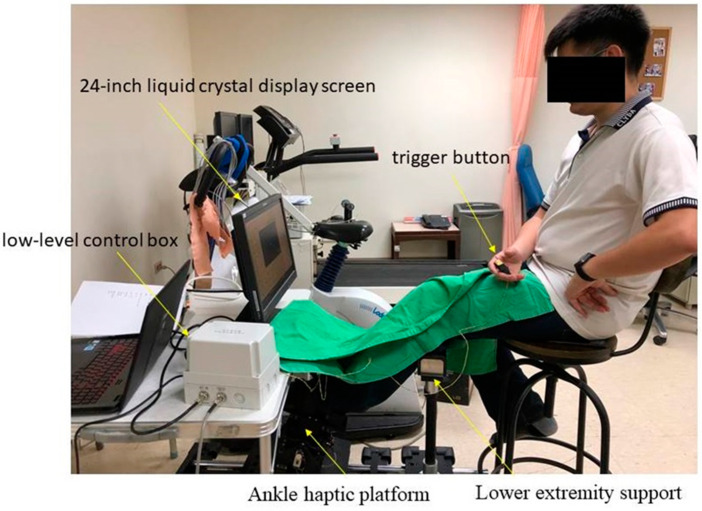
Experimental setting.

There are several small springs under the platform, which provide force feedback of multiple movements in tested muscles during muscle perception tests. A highly adjustable lower extremity support with Velcro^®^ was aligned with the ankle haptic platform. Each participant was asked to put their thigh on the lower extremity support and fix it with Velcro^®^ to avoid potential abnormal compensatory movements in the tested ankle joint (Figure 3). 

**Figure 4:**
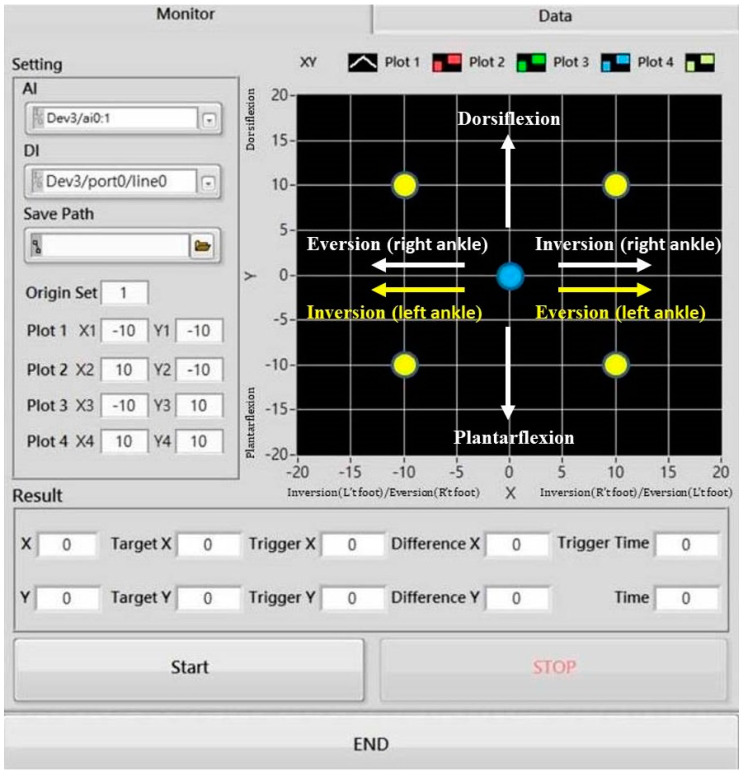
Control panel in LabVIEW for measuring muscle perceptions in four multiaxis test positions (plots 1–4).

All ankle movement data were amplified, filtered by low-level control box, and then collected and passed to a personal computer using a data acquisition system (USB-6003 Multifunction I/O and NI-DAQmx, National Instruments, Austin, TX, USA), and data were calculated and analyzed using LabVIEW (2015 edition, National Instruments, Austin, TX. USA). The self-developed control panel for measuring muscle perceptions was designed using LabVIEW to acquire data from the rotary potentiometers. The sampling rate was set to 1 kHz. The degree of ankle movement in the muscle perception tests was shown on the control panel on a 24-inch liquid crystal display screen in LabVIEW (Figure 4). 

Control panel for measuring muscle perceptions in four multiaxis test positions, including the positive data shown in X1–4 are inversion movements of the right ankle and eversion movements of the left ankle in degrees; the negative data shown in X1–4 are eversion movements of the right ankle and inversion movements of the left ankle in degrees; and the positive and negative data shown in Y1–4 are dorsiflexion and plantarflexion movements of both ankles in degrees (Figure 4).

### 2.4. Multiaxis Muscle Perception Tests in Both Ankles and Outcome Measurements

For the muscle perception tests in multiaxis movements, participants were asked to actively move their test ankle from a starting position (90° neutral position) to one of the specific multiaxis test positions with eyes open to watch the control panel to confirm whether the tested feet had achieved the specific test position, to hold that position for 10 s to sense and confirm the test position angle, and then move the tested ankles back to the starting position. Subsequently, the participants were asked to move their tested ankles to the same test position with their eyes closed and press a trigger button to confirm when they had reached the same position (Figure 3). To prevent potential learning effects with repeated measures, four test positions were randomly selected and tested twice in each participant. The joint position reproduction performance angles of the muscle perception tests in the sagittal and frontal planes were calculated for statistical analysis. Nonparetic ankle joints were tested first to familiarize each participant with the tests before the paretic limb was tested. One practice trial was performed before data collection. A 30–60-s resting interval was applied after each test to prevent muscle fatigue.

The parameters of direction error (DE), absolute error (AE), and variable error (VE) on multiple planes of the muscle perception test were calculated and analyzed to reflect the capability of perception by each muscle [27]. The DE in degrees indicated the difference between the test position and reproduced angle (collected angle value − target angle value) and showed the muscle’s sensory tendencies for achieving the test position [35,36,37,38]. AE was calculated to show the level of AE between the test position angle and reproduced angle (|angle value − target angle value|) and to indicate the muscle’s sensory accuracy of the test position [35,36,37,38,39]. VE between the obtained angle and test position angle was calculated using the root mean square error algorithm to indicate the consistency of a muscle’s perception performance of the test ankle [35,37]:

$$(\sqrt{{\left(\mathrm{collected}\;\mathrm{angle}\;\mathrm{value}\;-\;\mathrm{target}\;\mathrm{angle}\;\mathrm{value}\right)}^{2}}).$$

### 2.5. Clinical Motor and Functional Measurements

To understand the relationship of muscle perception deficits with motor and functional performances in the lower limbs of stroke patients, several clinical assessment scales that evaluate motor and functional performances with validity and reliability were employed, including the lower extremity portion of the Fugl-Meyer Assessment (FMA-LE); a balance evaluation included in the Postural Assessment Scale for Stroke Patients (PASS), Berg Balance Scale (BBS), Tinetti Performance-Oriented Mobility Assessment (POMA), and Timed Up and Go (TUG) test; daily functional evaluations involved in a 10-m walk test (10MWT). FMA-LE is the most common research and clinical tool for determining motor control in the paretic limbs of stroke patients [40,41,42]. The Barthel Index (BI) is also commonly used to understand the independent capabilities of individuals during daily activities and features excellent inter-rater reliability [43]. The PASS, BBS, POMA, TUG test, and postural and balance tasks are the most commonly used clinical assessment tools for precisely identifying functional performances with respect to posture control, balance, mobility, and risk of falls [44,45,46]. The 10MWT was employed in a previous study to validate walking mobility performance in patients with neurological diseases [47].

### 2.6. Statistical Analysis

Descriptive statistics was used to describe the basic demographic data of healthy participants and stroke patients. The normality of these data (DE, AE, and VE) was evaluated using the Shapiro-Wilk Test. Then, differences in muscle perception performance according to DE, AE, and VE for each muscle between the ankles in both healthy and stroke groups were determined using the paired-sample *t*-tests. The Mann-Whitney U test was performed to analyze differences among DE, AE, and VE values in each muscle between the healthy and stroke groups. Spearman correlation coefficient was applied to determine the relationship of the paretic ankle muscle’s perception variables (DE, AE, and VE) with motor and functional performances (FMA-LE, PASS, BBS, POMA, TUG test, and 10MWT). The α level of statistical significance was set at 0.05; SPSS version 17.0 software (IBM, Armonk, NY, USA) was used in this study.

## 3. Results

### 3.1. Muscle Perception Performances of Healthy and Stroke Groups

Results revealed that the VE scores of muscle perception for EDL, PL, and FDL in the dominant ankle were significantly lower than those in the nondominant ankle by 53.6% (*t* = −3.297, *p* = 0.003), 54.3% (*t* = −2.697, *p* = 0.014), and 55.9% (*t* = −2.583, *p* = 0.017), respectively (Table 2).

This indicated that the consistency of perception performances of muscles in the nondominant ankle was significantly poorer than that in the dominant ankle (Table 2). Results of the stroke group also revealed that VE scores of muscle perception in EDL in the nonparetic ankle were significantly lower than those in the paretic ankle by 59.9% (*t* = −2.995, *p* = 0.009), revealing that stroke decreased the consistency of the muscle perception performance in the paretic ankle compared with the nonparetic ankle (Table 3).

### 3.2. Stroke-Related Changes in Perception Performances of Muscles between Groups

**Figure 5:**
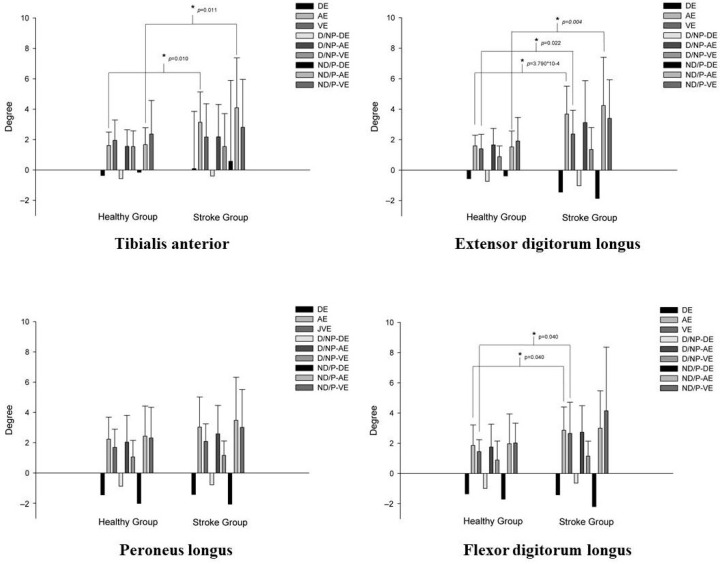
Differences in muscle perception performances in the four test positions between the healthy and stroke groups. DE, direction error; AE, absolute error; VE, variable error; P, paretic limb; NP, nonparetic limb; D, dominant limb; ND, nondominant limb.

This study also indicated stroke-related changes in perception performances in these muscles. For example, we found that the overall AE scores in TA in the healthy group were significantly lower than those in the stroke group by 48.7% (*t* = −2.857, *p* = 0.010, Cohen’s *d* = −0.938) (Figure 5).

Further, the results revealed that the AE scores of the nondominant ankles in the healthy group were significantly lower than those of the paretic ankles in the stroke group by 59.2% (*t* = −2.831, *p* = 0.011, Cohen’s *d* = −0.930) (Figure 5). Additionally, the results revealed that the overall AE and VE scores in EDL in the healthy group were significantly lower than those in the stroke group by 46.8% (*t* = −4.347, *p* < 0.001, Cohen’s *d* = −1.428) and 34.6% (*t* = −2.396, *p* = 0.022, Cohen’s *d* = −0.787), respectively (Figure 5). The results showed that the AE scores of the nondominant ankles in the healthy group were significantly lower than those of the paretic ankles in the stroke group by 63.9% (*t* = −3.297, *p* = 0.004, Cohen’s *d* = −1.083) (Figure 5). The results also indicated that the overall AE and VE scores in FDL in the healthy group were significantly lower than those in the stroke group by 35.9% (*t* = −2.134, *p* = 0.040, Cohen’s *d* = −0.701) and 22.6% (*t* = −2.208, *p* = 0.040, Cohen’s *d* = −0.725), respectively (Figure 5).

### 3.3. Relationships of Muscle Perception Performances with Clinical Motor and Functional Performances in the Stroke Group

We found that DE scores in TA of the paretic ankle joints were significantly correlated with FMA-LE (*p* = 0.04) and POMA scores (*p* = 0.011). AE (*p* = 0.04) and VE (*p* = 0.03) scores in EDL of paretic ankle joints were significantly correlated with 10MWT scores (*p* < 0.05). Additionally, DE scores in FDL of the paretic ankle were significantly correlated with FMA-LE (*p* = 0.04) and BI scores (*p* = 0.03), and AE scores in the FDL of the paretic ankle were significantly correlated with BI scores (*p* = 0.023) (Table 4).

## 4. Discussion

In this study, we aimed to evaluate stroke-related changes in muscle perceptions in multiple movements of the paretic ankle and their correlation with motor and function performance of lower limbs for people after stroke. Based on the multiaxis movements by muscles of the ankle joint, this is the first study to evaluate muscle perceptions in multiaxis movements, and results indicated stroke-related changes in muscle perceptions using a multiaxis ankle haptic interface. In most daily activities, perception feedback is provided by numerous receptors, especially in muscle spindles and Golgi tendon organ (GTOs) because changes in muscle tension and length occur when muscles voluntarily contract during movement [10,11,12]. However, stroke results in weakened muscles and abnormal muscle tone in paretic limbs [3,4,5,6], which could impact perceptions by specific muscles in the ankle joint. Based on the study findings, such physiological changes seem to indicate that interference with inputs to muscle spindles and GTOs from muscle tissues could consequently influence the perception-differentiating ability of the sensorimotor cortex, thereby rendering the brains of stroke patients unable to truly perceive inputs from paretic limbs [48].

### 4.1. Perception Performances in Multiaxis Movements of Muscles of Both Ankle Joints in the Healthy Group

We found that various test positions for different muscles resulted in different muscle perception performances in healthy participants. For example, in the healthy group, VE in EDL, PL, and FDL of the dominant ankle (0.89 ± 0.70, 1.06 ± 1.09, and 0.89 ± 1.26, respectively) were significantly lower than those of the nondominant ankle (1.92 ± 1.54, 2.32 ± 2.03, and 2.02 ± 1.32, respectively), which is in contrast to that in TA. These findings suggested that different movements could induce different sensory inputs to the central nervous system (CNS). There are two potential reasons to explain these differences in multiaxis movements resulting in differences in ankle muscle perception performance: (1) stretching of the skin and ligament and (2) co-contraction of multiple synergistic muscles on different axial movements. First, many ligaments surround the ankle joint, and these could be stretched during movement to provide multiple joint position inputs to the CNS [49,50]. Proprioceptive feedback and accurate joint perception information contributed by each ligament may differ. Therefore, specific ligaments may be stretched by different ankle movements to produce proprioceptive feedback to the CNS. Our results also revealed that muscle perception performance in the paretic ankle was significantly more varied than that in the nonparetic ankle during muscle perception tests in eversion from a dorsiflexion position, which could be attributed to the most common and sensitive ligaments around the ankle joint (calcaneofibular ligament and anterior and posterior talofibular ligaments); these ligaments were stretched and provided additional perception inputs to the unaffected brain, thereby helping the brain differentiate perception changes. Second, we based the EMG of the ankle musculature during gait cycle and clinical kinesiology performance and assessment reports of each muscle on multiaxis movements, and the four test positions chosen represented each muscle’s perception of the ankle joint [22,28]. However, in addition to these muscles, other muscles are involved in controlling ankle movements simultaneously, which might contribute to muscle perception feedback on various axes for different test positions [51]. For example, Tortora and Grabowski reported that EDL also contributes to ankle inversion with dorsiflexion movement; TA also contributes to ankle eversion with dorsiflexion movement; and the gastrocnemius and soleus muscles contribute to ankle inversion/eversion with plantarflexion (mainly contribute to ankle inversion with plantarflexion) [51].

### 4.2. Influence of Stroke on Ankle Muscle Perceptions in the Paretic Ankles in Stroke Patients

In addition to the results showing that VE of EDL for the paretic ankle was significantly higher (×2.5) than that for the nonparetic ankle, no significant differences in DE, AE, or VE in TA, PL, or FDL between the paretic and nonparetic ankles were observed. These findings are not surprising considering that recent studies also indicated that stroke patients could have bilateral perception deficits in both their paretic and nonparetic limbs and exhibit no significant differences in perception performances [52,53]. This phenomenon could be the result of a stroke causing injury to the perception integration and processing regions of the brain, including the primary motor and sensory cortex, supplementary motor area, cerebellum, and putamen [54,55].

Additionally, we found that the AE score of TA in the paretic ankle was higher than that in the nonparetic ankle by 1.9°, and VE scores of PL and FDL of the paretic ankle were significantly higher than those of the nonparetic ankle by 1.9° and 3.0°, respectively, in the stroke group. A previous study also revealed a similar finding and observed that AE scores in the paretic ankle were significantly higher than those in the nonparetic ankle and healthy young adults by 10° [56]. Furthermore, compared with a previous study that used a passive joint reproduction test under a non-weight-bearing condition, this study performed an active joint reproduction test under a partial weight-bearing condition (weight of the tested calf and foot, approximately 5% of the total body weight), which seemed to more precisely differentiate stroke-related changes in perception (1.9° vs. 10°; differentiating smaller changes in AE scores) [39,56]. This could be because of the involvement of muscles and generation of more perception-afferent impulses to the CNS during muscle-perception tests in multiaxis movements and under partial weight-bearing conditions.

### 4.3. Stroke-Related Changes in Ankle Muscle Perceptions between the Healthy and Stroke Groups

Our results indicated that compared with the healthy group, the stroke group exhibited poorer muscle perception accuracy (AE) in TA, showing that muscle perceptions in the nondominant ankles of the healthy group were significantly more precise than those in the paretic ankles in the stroke group. The results also indicated that the overall accuracy (AE) and consistency (VE) of muscle perception performances in EDL and FDL of the dominant and nondominant ankles in the healthy group were significantly higher than those of the paretic and nonparetic ankles in the stroke group. These findings could have resulted from central neurologic impairments and were similar to those of previous studies, indicating stroke-related decreases in the position perception in the upper and lower extremities of stroke patients [52,53,54]. This phenomenon could result from central neurologic impairments and causes abnormal muscle tone and muscle weakness in the paretic limbs and poorer perception inputs from muscles [5,6,7,8,9].

### 4.4. Relationships of Muscle Perceptions with Clinical Motor and Functional Performances in Stroke Patients

Our results showed that AE and VE of EDL during combined dorsiflex and eversion multiaxis movements in the paretic ankle were significantly positively correlated with the 10MWT score; this finding revealed that more accurate dorsiflex and eversion multiaxis movement in the paretic ankle was associated with greater walking speed and higher functional performance in the lower limbs. Additionally, the results also indicated that muscle perceptions play a critical role in dorsiflexion for functional performance during activities of daily living. For example, DE of TA (combined dorsiflexion and inversion multiaxis movement) of the paretic ankle and AE and VE of EDL (combined dorsiflexion and eversion multiaxis movement) in the paretic ankle were significantly correlated with POMA and 10MWT scores, thereby revealing that muscle perceptions of dorsiflexion were significantly correlated with ambulation capability. Nordin et al. also revealed that TA and EDL are the key muscles in activating ankle movements during walking [28], and POMA and 10MWT are commonly applied to validate the balance, gait, and walking performance in clinical settings; this study indicated that perception deficits of these muscles are correlated with motor and functional performances in stroke patients. Further, earlier studies confirmed that poorer perception performance in dorsiflexion could weaken balance and walking ability [57] and increase fall risk [58].

### 4.5. Study Limitations and Future Recommendations

This study evaluated muscle perceptions via active joint reproduction tests in multiaxis movements at the ankle joint to indicate stroke-related changes in muscle perception performances in post-stroke patients. However, most stroke patients have weakened muscle strength and abnormal muscle tone and synergy patterns, resulting in these patients not being able to follow study protocols and perform muscle perception tests for paretic ankle joints. Therefore, based on the critical criteria for stroke patient enrollment, only a few stroke patients met the inclusion criteria for participation in this study. We recommend that future studies recruit more participants for further investigation. In addition, active reproduction tests in multiaxis movements were used to evaluate each muscle’s perception in this study. This study also followed muscle contraction movements and inferred muscle perception deficits in multiaxis movements following stroke and their relationships with motor and functional performances. However, we found that there are other muscles that contribute to perception feedback during active joint reproduction tests, such as the tibia posterior and triceps surae. Therefore, it is difficult to eliminate such sensory feedback from other muscles, and future studies should also consider this aspect. We recommend that future studies need to use surface electromyography (sEMG) to further precisely clarify muscle perception performance in multiaxis movements at the ankle joint. This study indicated that muscle perception performances in the paretic ankle were correlated with motor and functional performances in the lower extremities of stroke patients, and we suggest that future studies should develop appropriate rehabilitation programs to improve muscle perceptions for these specific muscles, which may be helpful in enhancing the motor and functional recovery of paretic ankle joints of stroke patients. Furthermore, the multiaxis ankle-joint-perception measurement system was developed as a prototype, and all sensors and wires were exposed. Therefore, environment factors, such as moisture, temperature, and unintended impacts could affect the signal and should be considered in future clinical applications.

## 5. Conclusions

This study revealed stroke-related changes in the accuracy and consistency of ankle muscle perception performances in the paretic ankles of stroke patients. The results also indicated correlations of muscle perception performances with motor and functional performances by ankle joints in post-stroke patients. The self-developed system can provide valuable information to monitor stroke-related changes in the ankle muscle perception for people after stroke, which can help clinical therapists develop appropriate rehabilitation programs to improve muscle perceptions and enhance the motor and functional recovery in stroke patients.

## Figures and Tables

**Table 1:** Participant characteristics.

	Healthy Participants (*n* = 22)	Stroke Patients (*n* = 16)
Sex (male/female)	9/13	12/4
Age (years)	47.1 ± 25.0	55.5 ± 10.3
Height (cm)	162.4 ± 9.3	165.2 ± 7.1
Weight (kg)	58.5 ± 11.5	68.3 ± 8.44
Dominant or paretic leg (right/left)	24/1	10/6
Onset time (months)	-	27.5 ± 18.4
Lesion type (hemorrhagic/thrombotic)	-	6/10
Brunnstrom stage 4/5	-	6/10
FMA-LE	-	26.9 ± 6.5
BI	-	99.1 ± 2.0
BBS	-	49.9 ± 4.4
PASS	-	33.8 ± 1.4
POMA	-	9.8 ± 2.1
TUG (s)	-	22.3 ± 15.1
10MWT (s)	-	26.0 ± 19.6

FMA-LE, lower extremity portion of the Fugl-Meyer Assessment; BI, Barthel Index; BBS, Berg Balance Scale; PASS, Postural Assessment Scale for Stroke Patients; POMA, Tinetti Performance-Oriented Mobility Assessment; TUG, Timed Up and Go; 10MWT, 10-m walk test.

**Table 2:** Differences in muscle perception performance between the dominant and nondominant ankles of healthy participants (*n* = 22).

	Dominant Ankle	Nondominant Ankle	Mean Difference	95% CI	Statistical Value	*p* Value
TA						
DE	−0.57 ± 1.84	−0.14 ± 2.03	−0.43 ± 3.10	−1.805 to 0.944	*t*_(21)_ = −0.651	0.522
AE	1.56 ± 1.09	1.67 ± 1.11	−0.12 ± 1.33	−0.702 to 0.472	*t*_(21)_ = −0.408	0.688
VE	1.54 ± 1.03	2.37 ± 2.21	−0.82 ± 2.17	−1.785 to 0.137	*t*_(21)_ = −1.782	0.089
EDL						
DE	−0.73 ± 1.87	−0.38 ± 1.84	−0.36 ± 2.33	−1.388 to 0.676	*t*_(21)_ = −0.716	0.482
AE	1.66 ± 1.08	1.53 ± 1.04	0.13 ± 1.60	−0.579 to 0.838	*t*_(21)_ = 0.381	0.707
VE	0.89 ± 0.70	1.92 ± 1.54	−1.03 ± 1.46	−1.675 to −0.379	*t*_(21)_ = −3.297	0.003 *
PL						
DE	−0.87 ± 2.59	−2.01 ± 2.43	1.14 ± 3.25	−0.303 to 2.581	*t*_(21)_ = 1.642	0.115
AE	2.04 ± 1.77	2.43 ± 1.98	−0.39 ± 2.40	−1.458 to 0.674	*t*_(21)_ = −0.765	0.453
VE	1.06 ± 1.09	2.32 ± 2.03	−1.26 ± 2.19	−2.231 to −0.288	*t*_(21)_ = −2.697	0.014 *
FDL						
DE	−0.99 ± 2.12	−1.70 ± 2.21	0.71 ± 2.26	−0.292 to 1.711	*t*_(21)_ = 1.473	0.156
AE	1.75 ± 1.52	1.97 ± 1.97	−0.22 ± 2.25	−1.218 to 0.777	*t*_(21)_ = −0.459	0.651
VE	0.89 ± 1.26	2.02 ± 1.32	−1.12 ± 2.04	−2.029 to −0.219	*t*_(21)_ = −2.583	0.017 *

TA, tibialis anterior; EDL, extensor digitorum longus; PL, peroneus longus; FDL, flexor digitorum longus; CI, confidence interval; DE, direction error; AE, absolute error; VE, variable error. * *p* < 0.05.

**Table 3:** Differences in muscle perception performances between the paretic and nonparetic ankles in stroke patients (*n* = 16).

	Nonparetic Ankle	Paretic Ankle	Mean Difference	95% CI	Statistical Value	*p* Value
TA						
DE	−0.394 ± 3.07	0.580 ± 5.31	−0.974 ± 4.34	−3.286 to 1.338	*t*_(15)_ = −0.898	0.383
AE	2.188 ± 2.12	4.092 ± 3.28	−1.904 ± 3.82	−3.942 to 0.134	*t*_(15)_ = −1.991	0.065
VE	1.541 ± 2.16	2.810 ± 3.15	−1.269 ± 3.18	−2.963 to 0.426	*t*_(15)_ = −1.595	0.131
EDL						
DE	−1.021 ± 4.10	−1.850 ± 5.05	0.829 ± 6.03	−2.383 to 4.041	*t*_(15)_ = 0.550	0.591
AE	3.123 ± 2.75	4.240 ± 3.17	−1.117 ± 4.68	−3.608 to 1.375	*t*_(15)_ = −0.955	0.355
VE	1.361 ± 1.44	3.400 ± 2.53	−2.035 ± 2.72	−3.482 to −0.587	*t*_(15)_ = −2.995	0.009 *
PL						
DE	−0.774 ± 3.16	−2.060 ± 4.07	1.286 ± 4.25	−0.978 to 3.549	*t*_(15)_ = 1.210	0.245
AE	2.591 ± 1.87	3.485 ± 2.85	−0.894 ± 2.74	−2.353 to 0.566	*t*_(15)_ = −1.305	0.212
VE	1.163 ± 0.96	3.017 ± 2.50	−1.854 ± 3.00	0.751 to −3.454	*t*_(15)_ = −2.469	0.026
FDL						
DE	−0.635 ± 3.26	−2.191 ± 3.25	1.555 ± 4.338	−0.756 to 3.867	*t*_(15)_ = 1.434	0.172
AE	2.730 ± 1.76	2.996 ± 2.47	−0.266 ± 2.977	−1.852 to 1.320	*t*_(15)_ = −0.357	0.726
VE	1.150 ± 0.99	4.155 ± 4.21	−3.005 ± 4.507	−5.406 to −0.603	*t*_(15)_ = −2.667	0.018

TA, tibialis anterior; EDL, extensor digitorum longus; PL, peroneus longus; FDL, flexor digitorum longus; CI, confidence interval; DE, direction error; AE, absolute error; VE, variable error. * *p* < 0.05.

**Table 4:** Correlations of muscle perception performances with clinical motor and functional outcome measurements (*n* = 16).

	FMA-LE		PASS		BBS		POMA		TUG		10MWT		BI	
	*r* Value	*p* Value	*r* Value	*p* Value	*r* Value	*p* Value	*r* Value	*p* Value	*r* Value	*p* Value	*r* Value	*p* Value	*r* Value	*p* Value
TA														
DE	−0.509	0.044 *	−0.195	0.470	−0.334	0.206	−0.619	0.011 *	0.464	0.070	0.486	0.056	−0.388	0.138
AE	−0.036	0.894	0.283	0.288	0.251	0.349	0.059	0.827	0.035	0.897	0.123	0.649	−0.305	0.250
VE	0.037	0.893	0.131	0.629	−0.036	0.895	−0.226	0.401	−0.028	0.919	0.189	0484	−0.367	0.162
EDL														
DE	−0.319	0.229	0.159	0.557	−0.016	0.953	−0.307	0.248	0.360	0.171	0.460	0.073	−0.413	0.112
AE	−0.246	0.358	−0.106	0.697	−0.167	0.537	−0.065	0.810	0.436	0.091	0.515	0.041 *	−0.341	0.196
VE	−0.037	0.891	−0.039	0.886	−0.295	0.267	−0.383	0.143	0.363	0.167	0.557	0.025 *	−0.315	0.234
PL														
DE	0.162	0.548	0.105	0.698	0.049	0.856	0.111	0.681	−0.187	0.489	−0.199	0.460	0.118	0.663
AE	−0.310	0.242	−0.023	0.934	−0.054	0.842	−0.192	0.475	0.146	0.589	0.168	0.535	−0.339	0.200
VE	0.065	0.811	−0.343	0.193	−0.251	0.349	−0.311	0.242	0.404	0.121	0.361	0.170	0.416	0.109
FDL														
DE	0.519	0.039 *	0.332	0.208	0.206	0.444	0.172	0.525	0.003	0.992	0.082	0.764	0.551	0.027 *
AE	−0.403	0.122	−0.055	0.838	0.086	0.752	0.000	0.999	−0.183	0.498	−0.201	0.456	−0.562	0.023 *
VE	0.478	0.061	0.275	0.303	0.315	0.235	0.277	0.299	−0.135	0.618	−0.009	0.974	0.444	0.085

FMA-LE, Lower Extremity portion of the Fugl-Meyer Assessment; PASS, Postural Assessment Scale for Stroke Patients; BBS, Berg Balance Scale; POMA, Tinetti Performance-Oriented Mobility Assessment; TUG, Timed Up and Go; 10MWT, 10-m walk test; BI, Barthel Index; TA, tibialis anterior; EDL, extensor digitorum longus; PL, peroneus longus; FDL, flexor digitorum longus; DE, direction error; AE, absolute error; VE, variable error. * *p* < 0.05.

## Data Availability

Please contact the corresponding author for data requests.
